# Summary and conclusions, and abbreviations and acronyms

**DOI:** 10.1002/acm2.13846

**Published:** 2023-01-27

**Authors:** Wayne D. Newhauser, Jacqueline P. Williams, Michael A. Noska, Caridad Borrás, E. Vincent Holahan, Shaheen A. Dewji, Thomas E. Johnson, Jerry W. Hiatt, John W. Poston, Nolan Hertel, Dustin A. Gress, Michael D. Mills, David W. Jordan, Steven G. Sutlief, Melissa C. Martin, Edward Jackson, Edward I. Bluth, Donald P. Frush, M. Elizabeth Oates, Jeanne LaBerge, Hubert Y. Pan, Seth A. Rosenthal, Lawrence W. Townsend, Lori Brady, Janice Lindegard, Howard L. Hall, Elizabeth McAndrew‐Benavides, Eric Abelquist, Mitchell S. Anscher, Marcelo Vazquez, Amy Kronenberg, Jeffrey S. Willey, Theodore Lawrence, Gayle E. Woloschak, Brian Marples, Rosemary Wong, Michael Story, Roger W. Howell, Tom K. Hei, Sergey Y. Tolmachev, John D. Auxier, Thomas L. Rucker, Mikael Nilsson, Ralf Sudowe, Brian A. Powell, Mark P. Jensen

**Affiliations:** ^1^ Department of Physics & Astronomy Louisiana State University and Mary Bird Perkins Cancer Center Baton Rouge Louisiana USA; ^2^ University of Rochester Medical Center Rochester New York USA; ^3^ U.S. Food & Drug Administration Silver Spring Maryland USA; ^4^ Georgetown University Washington District of Columbia USA; ^5^ U.S. Nuclear Regulatory Commission Washington District of Columbia USA; ^6^ Department of Nuclear Engineering Texas A&M University College Station Texas USA; ^7^ Colorado State University Fort Collins Colorado USA; ^8^ Nuclear Energy Institute Washington District of Columbia USA; ^9^ Texas A&M University College Station Texas USA; ^10^ Georgia Institute of Technology North Avenue Atlanta Atlanta Georgia USA; ^11^ American College of Radiology Reston Virginia USA; ^12^ James Graham Brown Cancer Center Louisville Kentucky USA; ^13^ University Hospitals Cleveland Medical Center Cleveland Ohio USA; ^14^ Banner MD Anderson Cancer Center Phoenix Arizona USA; ^15^ Therapy Physics Inc. Gardena California USA; ^16^ University of Wisconsin Madison Wisconsin USA; ^17^ Ochsner Clinic Foundation New Orleans Louisiana USA; ^18^ Duke University Childrens Health Center Durham North Carolina USA; ^19^ University of Kentucky Lexington Kentucky USA; ^20^ University of California San Francisco San Francisco California USA; ^21^ Anderson Cancer Center Houston Texas USA; ^22^ Sutter Radiation Oncology Center Roseville California USA; ^23^ University of Tennessee, Zeanah Engineering Complex Knoxville Tennessee USA; ^24^ American Nuclear Society La Grange Park Illinois USA; ^25^ University of Tennessee, Zeanah Engineering Complex Knoxville Tennessee USA; ^26^ Electric Power Research Institute Palo Alto California USA; ^27^ Oak Ridge Associated Universities Oak Ridge Tennessee USA; ^28^ Virginia Commonwealth University School of Medicine Richmond Virginia USA; ^29^ Loma Linda University Loma Linda California USA; ^30^ Lawrence Berkeley National Laboratory Berkeley California USA; ^31^ Wake Forest School of Medicine Winston‐Salem North Carolina USA; ^32^ University of Michigan–University Hospital Ann Arbor Michigan USA; ^33^ Northwestern University Chicago Illinois USA; ^34^ National Cancer Institute Rockville Maryland USA; ^35^ The University of Texas Southwestern Dallas Texas USA; ^36^ Rutgers New Jersey Medical School Newark New Jersey USA; ^37^ Columbia University New York New York USA; ^38^ Washington State University Richland Washington USA; ^39^ University of Tennessee, Ferris Knoxville Tennessee USA; ^40^ Leidos Reston Virginia USA; ^41^ University of California Irvine Irvine California USA; ^42^ Clemson University Rich Lab Anderson South Carolina USA; ^43^ Colorado School of Mines Golden Colorado USA

**Keywords:** professional radiation workforce

## 8.1 CURRENT STATUS AND FUTURE OUTLOOK OF THE WORKFORCE IN THE UNITED STATES

Each team consisted of subject matter experts in the respective profession; they considered data from the literature and other sources, such as surveys conducted by, and membership trends in, relevant professional societies. However, almost without exception, it was apparent that there are significant limitations in precisely determining the current status and temporal trends of the professions considered. The reasons for this include: several of the professions have few to no means of surveilling their workforce; many lack well‐defined training programs; and basic concepts and terminology for professional standards and qualifications are frequently ill‐defined, including a proliferation of titles for the same position (e.g., radiation protection manager, radiation protection expert, radiation safety officer, and other titles conflated with health physicist). Therefore, all conclusions and recommendations, unless otherwise noted, represent the consensus expert opinions of the authors.

Overall, the authors found that the current status and future outlook of the professions involved in radiation protection varied considerably dependent on specialty, subspecialty, and other factors. These are summarized in Table 1 and below; additional details and supporting sources may be found in Chapters 2 through 7:

- Health physics: The health physics workforce is currently deemed adequate to meet routine needs, except those likely to be associated with a major radiological incident. However, the size of the workforce is in sustained decline due to a variety of factors, including reduced demand from the civilian nuclear power industry, a major employer of health physicists. The educational pipeline also is under stress, with declining trends in admissions, graduations, and the number of viable degree programs. Concerns also were raised with respect to worker retirement and the related loss of experience. Therefore, the future adequacy of the health physics workforce cannot be predicted due to uncertainties in the projected supply of, and demand for, workers.
- Medical physics: The medical physics workforce is currently deemed adequate in size, although there are indications of shortages in some subspecialties, such as diagnostic imaging and nuclear medicine. Although the education pipeline is seen as adequate for current requirements, this may not be true in the future due to limitations on the number of available residency training positions. As a result, there is moderate uncertainty over the future adequacy of the medical physics workforce due to uncertainties in both the supply of new graduates, as well as an unpredictable demand for medical physics services.
- Medicine: Currently, there is a shortage of radiologists in many radiologic specialties and subspecialties. In addition, changes in workforce practice and conditions likely have contributed to an increased incidence of burnout and an apparent growing interest in part‐time, versus full‐time, work. However, in the future, this shortage of radiologists may be counterbalanced by further changes in practice and/or technologies, such as the use of artificial intelligence and deep learning, telecommuting and wider adoption of telemedicine, especially in rural areas. In contrast, technological and practice changes appear to be decreasing the overall need for radiation oncologists, possibly leading to a shortage of positions available to residents entering the workforce in the near future. This trend may be countered by fully certified medical professionals moving to currently under‐served geographic areas.
- Nuclear engineering: The present nuclear engineering workforce is considered to be adequate, although there are concerns regarding the loss of available knowledge and experience with worker retirements. The education pipeline is currently sufficient and robust, however, there are concerns that include possible insufficiency of entry‐level positions for future graduates if the recent trend of nuclear power plant closures continues. Development of a diverse workforce, such as increasing the inclusion of women and minorities, remains challenging.
- Radiation biology: The radiation biology workforce has undergone a dramatic decline in the number of qualified personnel, the result of ongoing retirements coupled with a failure to replace these workers, as well as decreased research funding. These same factors also have introduced significant stress into the education pipeline, which has seen the closure of all but a few specialized training programs. The future outlook for this profession is poor, a situation that, unless rectified, will not only affect the progress of radiobiological research, but also the education of radiation oncology and radiology residents.
- Radio‐ and nuclear chemistry: The workforce in radiation chemistry and nuclear chemistry is small, diverse, multidisciplinary, and believed to be generally adequate to meet current needs, in particular, due to a recent resurgence. However, due to a decrease in educational opportunities, it is unclear if there is sufficient capacity to replenish the aging workforce in the future.

**TABLE 1:** Select characteristics of the professional radiation workforce in the United States by discipline, as determined in 2018

Aspect	Health physics	Medical physics	Medicine	Nuclear engineering	Radiation biology	Radiation and nuclear chemistry
Size (number of workers)	3200–7000	8000	37 600 (34 000 radiologists; 3600 radiation oncologists)	18 000	∼500	Estimate not available
Trends in workforce size	Shrinking	Growing; shortages in some subspecialties	Changing practices in radiology and radiation oncology affecting workforces	Slight growth; aging workforce lacking in diversity	Shrinking; shortages due to aging workforce, failure to replace	Shrinking; shortages due to aging workforce
Factors driving future trends	Closure of power plants	Increasing demand due to population growth/aging	Aging/retirements, employment choices (full vs. part‐time), use of AI	Increase in nonpower applications (e.g., nuclear security)	Aging/retirements, reduced funding	Aging/retirements, reduced funding
State of education/training	Small capacity; risk of program closure	Limited residency positions may affect the future pipeline	Adequate capacity	Adequate capacity	Complete loss of training programs	Risk of future inability to maintain the workforce

Table 1 lists selected aspects of the six professions considered in this report.

## 8.2 IMPLICATIONS FOR FUTURE INADEQUACIES IN THE WORKFORCE

Implications for workforce shortages are detailed in Chapters 2–7, and are summarized below. Of note, this list is based on the consensus expertise and experience of the individual writing teams, whose members are well‐placed experts in their respective fields.

- Health physics: A shortage of health physicists would lead to a detrimental impact on multiple services, including the nation's ability to provide radiation environmental monitoring and remediation, engage in emergency responses, maintain radiation safety standards within military and radionuclide production facilities, as well as overseeing selected safety aspects of the diagnostic and therapeutic uses of radiation in medicine. Thus, shortages in the health physics workforce will have a direct impact on regulatory compliance, the nation's defense capabilities, and worker and general population safety.
- Medical physics: A shortage of medical physicists would affect those areas of health practice where their expertise is a requirement for patient throughput, likely leading to an increase in the time intervals between diagnosis and the initiation of radiation treatment (most commonly for cancer), reduced quality and safety of clinical procedures, delayed implementation of new technologies, increased labor costs, and so forth. Thus, a decline in the medical physics workforce would have a direct impact on medical practice and the nation's health.
- Medicine: Shortages among the various radiation medical professions would result in increased challenges for both the timeliness and accuracy in the interpretation of diagnostic images, as well as treatment decision‐making and delivery, severely impacting patient outcomes. However, the risk of inadequate medical workforces in either radiology or radiation oncology is unclear due to ongoing changes in practice and technologies.
- Nuclear engineering: A shortage of nuclear engineers would likely accelerate the closure of nuclear power plants and reduce capabilities in nuclear security and nonproliferation, having a direct impact on national and energy security.
- Radiation biology: A shortage of radiation biologists would limit future progress in several medical fields, especially countermeasure development and radiation oncology, where the advancement of physical technologies now requires biological input. The educational needs of residents in some fields are already affected by the loss of trained radiobiology teachers. Thus, a shortage of radiobiologists would negatively affect the nation's defense capabilities, medical training, and practice.
- Radio‐ and nuclear chemistry: Shortages of workers within the radio‐ and nuclear chemistry workforces would affect multiple areas of national importance, including nuclear power generation, nuclear forensics, homeland defense, and medical applications. Thus, a shortage in the radio‐ and nuclear chemistry workforce will have a direct impact on the nation's defense capabilities, energy network, and medical practice.

## 8.3 PROFESSION‐SPECIFIC CONCERNS REGARDING ADEQUACY OF THE WORKFORCE IN THE FUTURE

The teams identified several profession‐specific concerns regarding the future workforces, which are summarized here and detailed in Chapters 2 through 7.

- Health physics: There is a decreasing number of admissions to health physics graduate programs, especially PhD programs, linked to declining interest from prospective students. In response, the number of degree‐granting programs is declining. There also are concerns regarding the availability of entry‐level jobs and clear career paths.
- Medical physics: Currently, the number of graduates from degree programs substantially exceeds admission levels to post‐graduate training programs since clinical residency training capacity is limited, especially in diagnostic physics and nuclear medical physics.
- Medicine: Burnout and early retirement of radiologists are diminishing the current workforce, stressing the pipeline. In contrast, due to changes in practice, the pipeline of radiation oncology trainees may exceed demand in the near future. It is uncertain whether this latter trend can be countered by medical professionals moving into “underserved” geographic areas.
- Nuclear engineering: The nuclear engineering workforce may require additional supply capacity should policy makers gain an increased appreciation of the benefits of clean, nuclear energy production as part of combating climate change and global supply fluctuations.
- Radiation biology: The loss of specialized training programs has had a detrimental effect on the profession. Identifiable career paths for those entering the field are lacking due to the reduction in available positions in academic and medical departments, partly related to declines in federal funding for both education and research.
- Radio‐ and nuclear chemistry: An increased need for nuclear medicine and isotope production appears likely to create future shortages of radio‐ and nuclear chemists.

## 8.4 COHERENCE WITH PREVIOUS REPORTS

The NCRP sponsored a workshop in 2013 to evaluate whether there was a sufficient number of radiation professionals, currently and into the future, that could support the various radiation disciplines essential to meet national needs (https://ncrponline.org/wp‐content/themes/ncrp/PDFs/WARP_Workshop_Summary.pdf). This effort led to a four‐page statement entitled “Where are the Radiation Professionals” (WARP) (Ncrp, 2015) that warned that “the country is on the verge of a severe shortfall of radiation professionals such that urgent national needs will not be met.” The statement identified mixed findings of adequacy of the workforces in the short term (5–10 years), but also projected insufficient numbers of workers into the long term (10–20 years). The immediate shortfall was attributed to an ongoing wave of retirements of baby‐boomers, declining enrollment in STEM degree programs, and other factors. To mitigate against the risk of an inadequate workforce, the statement recommended: adequate funding for education, training, and research; increased coordination of the federal government's civil service to support such efforts; and monitoring and advocacy.

Nine years have passed since the WARP statement was published. The suggestion of mixed short‐term (5–10 years) adequacy in the professional workforces has been broadly confirmed by the findings described in this issue. However, importantly, it was apparent that insufficient data were available in order to reliably predict the workforce status in the long term (10–20 years), a task that was further complicated by any additional impact from the COVID‐19 pandemic. Indeed, a major finding of the authors was the large number of gaps in the state of knowledge of several workforces. Qualitatively, this review reveals that many of the conceptual concerns expressed in the WARP statement remain relevant today, such as a looming wave of retirements of baby boomers, and insufficient capacity of training and education programs to produce new workers.

This review differs from the WARP statement in its organizational structure and depth. Specifically, it systematically and separately reviewed each of the six selected professions, including respective subspecialties. Attention was paid to providing background information, such as definitions of each profession, education, and training pathways, and placed additional emphasis on identifying specific examples of interdependencies of the radiation professions. These and other considerations are essential to understanding the professional radiation workforces and to inform recommendations to ensure their future adequacy. The overall approach embodied in this report may serve as an organizational framework for future workforce studies in the area, adding to the base of evidence that can inform decision‐making by the professions, employers, policy makers, and other stakeholders.

## 8.5 DISCUSSION

Overall, the authors found the available literature and data on the various radiation workforces to be insufficient to draw conclusions with high levels of certainty. Even basic data, such as the number currently working in each profession, were in some cases unavailable or incomplete. Indeed, in many of the professions, basic definitions and terminology used to describe qualifications and career stages were either lacking, difficult to find, or open to interpretation. Therefore, recommendations were based on the expertise and experience of the respective writing teams. Issues of concern were identified, with some specific to individual radiation professions (detailed in Chapters 2–7), although a few were common to all, including attrition, limited elasticity in the supply of workers, and limited financial resources.

The recommendations below are consensus opinions on actions needed to ensure that the radiation professions considered will be able to meet the nation's future needs. The authors intentionally declined to recommend detailed methods, timelines, responsibilities of individual organizations, and funding sources, considered outside the scope of this review.

The authors recommend the following actions, which apply to all radiation professions considered in this review:

1. Initiating, supporting, or enhancing annual surveillance of radiation professionals in all relevant workforces. These data comprise an important evidence base for decision‐making by policy makers, employers, and other stakeholders.
2. Fostering cooperation, coordination, and harmonization among the professions. Since each of the described professions are focused on radiation utilization, delivery, and/or exposure, in particular with respect to radiation protection, efficient and appropriate regulation requires expertise from many, if not all, of the radiation professions.
3. Advocacy for generating sustainable funding in both higher education and research in the radiation sciences should be supported. Significantly, improved research funding levels are needed to stabilize a professional critical mass in some of the major radiation disciplines (e.g., radiation biology, radiation chemistry). Since the full spectrum of radiation sciences contributes towards the training and education of all fields, a critical mass of teachers/trainers and mentors must be maintained to enable viable educational pipelines of future professional workers; thus, support for research and education is interdependent. Because of the long lag time between matriculation in an education program and attainment of needed professional competencies, planning exercises may need to be conducted to ensure the adequacy of future workforces.
4. Outreach activities to attract future workers must be developed. Currently, this is of increased importance because of the imminent attrition of large proportions of the workforce who are reaching retirement age. Efforts should include public outreach, since a greater awareness of the benefits of the radiation sciences is needed at all societal levels. Collaborative partnerships between academia, national laboratories, and industry must be strengthened.

The authors recommend the following profession‐specific actions (see Chapters 2–7 for further details):

- Health physics: The supply of workers entering the profession needs to be stabilized by supporting academic programs. This will need to be coupled with more accurate projections of future workforce needs.
- Medical physics: Projections of demand for future workers must be improved, taking into account uncertainties in potential demand due to growth and aging of the population and changes in the nation's health care laws and policies.
- Medicine: A new national group, committee, or commission should be developed which can make national recommendations regarding the appropriate number of trainees in all of the radiation medical professions. Prospective medical students and educators should be informed about opportunities available in the radiation medicine sectors, in particular in those subspecialties where there are worker shortages.
- Nuclear engineering: Actions at the national and state levels are needed to ensure an adequate supply of nuclear engineers to meet future demands for workers in electrical power generation, isotope production, defense, and other areas of importance. Requisite actions should be targeted at outreach (public and academic) and improved education pipelines.
- Radiation biology: Specialized training programs must be re‐established so as to increase the supply of future radiation biologists. Academic institutions with radiation oncology and other related radiation science departments should be encouraged to increase the hiring of trained radiation biologists, with institutional commitments to maintaining positions and providing succession plans for those who are retiring. These are needed to provide clear career paths for new entrants into the field.
- Radio‐ and nuclear chemistry: Academic programs in nuclear and radiochemistry must be sustained, and long‐term support for radiochemistry workforce education must be provided by federal agencies. If necessary, on‐the‐job training of graduates with chemistry and related degrees must be supported in order to facilitate knowledge transfer.

## AUTHOR CONTRIBUTIONS

All the authors listed have contributed directly to the intellectual content of the manuscript. Wayne Newhauser and Jacqueline Williams drafted the manuscript.

## CONFLICT OF INTEREST

The author declares that there is no conflict of interest.

